# Clinical and Echocardiographic Follow-up after Successful Percutaneous Transvenous Mitral Commissurotomy

**DOI:** 10.7759/cureus.1726

**Published:** 2017-09-29

**Authors:** Imran Khan, Bakhtawar Shah, Mohammad Habeel Dar, Adnan Khan, Malik Faisal Iftekhar, Abdul Sami

**Affiliations:** 1 Cardiology/cardiac Electrophysiology, Hayatabad Medical Complex Peshawar; 2 Cardiology, Hayatabad Medical Complex Peshawar; 3 Cardiology, Moulvi Ameer Shah Hospital Peshawar; 4 House Officer, Rehman Medical Institute, Peshawar

**Keywords:** mitral stenosis, ptmc, long-term survival

## Abstract

Background

The objective of the study was to determine the long-term outcomes, including mitral restenosis and regurgitation, after successful percutaneous transvenous mitral commissurotomy (PTMC).

Methods

This cross-sectional prospective study was conducted at the cardiology department of Lady Reading Hospital, Peshawar, Pakistan, from January 2007 to December 2009. A total of 84 patients were followed up for a period of 96 months. Pre and post percutaneous transvenous mitral commissurotomy echocardiography was done on the mitral valve area (MVA) using two-dimensional (2D) and color doppler echocardiography. Patients who had successful PTMC were followed up for MVA loss, mitral regurgitation (MR), and cardiac death. SPSS Software (IBM SPSS Statistics for Windows, Version 22.0, Armonk, NY: IBM Corp.; 2013) was used for data analysis.

Results

Of the 84 patients, 21 were male, and 63 were females. The mean age was 35 ± 11 years. After PTMC, the mean valve two-dimensional area increased from 0.84 ± 0.13 to 1.83 ± 0.49 cm^2^ (p value <0.001). MR was mild in 49 patients (62.8%), moderate in 27 patients (34.6%), and severe in two patients (2.6%). Good results were achieved in 60 (71.4%). Patients with good results were younger (24 ± 4), and had a relatively lower Wilkin's score, with a mean value of (8.4 ± 2.8). Follow-up events were death in six patients, mitral valve replacement (MVR) in 10 patients, and restenosis in seven patients. The Kaplan-Meier curve was used for the follow-up end points. Patient who required PTMC for mitral restenosis survived for a longer time than those requiring MVR, and those who had cardiac death due to severe pulmonary hypertension or heart failure.

Conclusion

Patients who had favorable Wilkin’s score and underwent PTMC for severe symptomatic mitral stenosis had better event-free survival in the long term follow-up.

## Introduction

In the western world, the incidence of rheumatic fever is decreasing, but it is still a common problem in developing countries like Pakistan with a prevalence rate of 22/1,000 population. Certain studies have reported the incidence of rheumatic fever and rheumatic heart disease (RHD) to be as high as 206/100,000 and 18.6/1000 respectively in different geographical areas of Pakistan [1-2].

Patients with acute rheumatic fever are asymptomatic initially, until severe mitral stenosis (MS) develops and patients become symptomatic. It then takes approximately 10 to 15 years to develop severe symptoms i.e. New York Heart Association (NYHA) Class IV symptoms and thus needs intervention. Patients with MS with favorable anatomy are treated with percutaneous approach. Patients with unsuitable anatomy, high Wilkin's score or moderate to severe mitral regurgitation (MR) are treated with mitral valve surgery.

Percutaneous transvenous mitral commissurotomy (PTMC) was first introduced by Inoue in 1982, and since then it is the preferred non-surgical alternative for severe symptomatic mitral stenosis [3]. Currently for patients with rheumatic heart disease and symptomatic severe mitral stenosis, PTMC is considered a safe and a standard procedure [4-5]. Post-PTMC, most patients with severe symptomatic mitral stenosis improve clinically and hemodynamically. Various studies have reported immediate good results [6-8]. There are only few studies in literature with longer follow-up [9-11].

However, no study is available in Pakistan with long-term follow-up after successful mitral balloon valvuloplasty. The objective of the present study was to determine long-term clinical and echocardiographic follow-up in patients with severe symptomatic mitral stenosis.

## Materials and methods

From January 2007 to December 2009, a consecutive series of 100 patients with mitral stenosis (mean age 25 ± 11 years) underwent percutaneous transvenous mitral commissurotomy at the Cardiology Department of Lady Reading hospital, Peshawar in Pakistan. Out of the 100 patients discharged after successful procedure, 16 were lost to follow-up. The remaining 84 (84% of the eligible) were followed up for 96 months, and are the subjects of this study.

Indication of percutaneous transvenous mitral commissurotomy included symptomatic severe mitral stenosis, less than moderate MR, and no left atrial or left atria appendage clot. Patients who had previous surgery or PTMC were not included in this study. That data was collected electronically and was recorded on a pre-formed proforma. The clinical status was determined by the New York Heart Association (NYHA) functional class. An echo-Doppler study was performed before and after 24 hours of conducting PTMC in all patients. Evaluations included mitral valve area (using planimetry method by two-dimensional (2-D) echocardiography and/or the Doppler pressure half-time method), echocardiographic score (Wilkin's score) [12], and MR estimation by color-Doppler visual method (graded from no MR to severe regurgitation). All patients underwent transesophageal echocardiography one day before the procedure to rule out left atrium or left atrial appendage thrombosis. The study protocol was approved by the ethical committee of our center.

Percutaneous transvenous mitral commissurotomy was performed via antegrade transvenous approach with the Inoue balloon, according to the standard protocol of our center. Balloon size was selected according to the formula (height in centimeters/10 + 10). A left ventriculogram was performed before and after the last balloon inflation to quantitate the amount of MR. Angiographic MR severity was graded from no mitral regurgitation to severe regurgitation.

A successful PTMC was defined by a mitral valve area (MVA) > 1.5 cm^2^ and a MR of moderate or less. Symptomatic improvement after PTMC was defined as ≥ 1 NYHA functional class dyspnea reduction, with a post-percutaneous transvenous mitral commissurotomy NYHA functional class dyspnea ≤ 2 at two month. Follow-up symptomtic impairment was considered as dyspnea worsened ≥ 1 NYHA class or NYHA class ≥3. Follow-up end points were cardiac death, mitral valve replacement (MVR), repeat PTMC for restenosis, and symptomatic evaluation according to NYHA functional classification of dyspnea in congestive heart failure. Restenosis was defined as loss of 50% of the initial gain in mitral valve area, or MVA <1.5 cm^2^.

All patients were followed up by outdoor patient department visits at six months, and then yearly after the initial PTMC. Patients were monitored for adverse events during the follow-up period. When the patient was lost to follow-up, the family was contacted.

Continuous variables were calculated as mean ± standard deviation. The categorical data were recorded as percentages. P-value of ≤ 0.05 was considered significant. Discrete data were compared by chi-square analysis, and comparisons before and after PTMC were made by using paired Student’s two-tailed t-test. Event-free survival rate for end points was determined with the Kaplan-Meier curve.

## Results

The baseline characteristics and initial results are shown in Table 1. Mean diameter of Inoue balloon was 28.6 ± 2.3 mm. After PTMC, mean mitral valve (calculated by two-dimentional echocardiography) increased from 0.84 ± 0.13 to 1.83 ± 0.49 cm^2^ (p-valve <0.001). Mean MVA (calculated by color Doppler method) increased from 0.91 ± 0.13 to 1.94 ± 0.45 cm^2^ (p-valve <0.001). Mean gradient decreased from 16 ± 5.2 to 6 ± 2.2 mmHg (p valve < 0.001). MR was mild in 49 (62.8%), moderate in 27 (34.6%) and severe in two (2.6%) patients. Good results were achieved in 60 (71.4%) patients. Patients with good results were younger (24± 4 years) and had a relatively lower Wilkin's score with a mean value of (8.4 ± 2.8). Those who developed mitral regurgitation were younger (25 ± 3 years) but the pre-PTMC area was smaller 0.5 ± 0.1 as shown in Table 1.

**Table 1:** Clinical and echocardiographic data of the patients before and after PTMC PTMC = Percutaneous transvenous mitral commissurotomy NYHA = New York Heart Association

	Pre-PTMC	Post-PTMC	P- value
Age in years (mean ± SD)	35 ± 11		
Male gender	21 (25%)		
Female gender	63 (75%)		
Sinus rhythm	58 (74.3)		
Left atrial diameter (cm^2^)	4.8 ± 0.8	4.6 ± 0.7	
2D Mitral area (cm^2^)	0.84 ± 0.13 (0.5 - 1.10)	1.83 ± 0.49 (1.10 - 3.50)	<0.001
Doppler Mitral area (cm^2^)	0.91 ± 0.13 (0.7 – 1.20)	1.94 ± 0.45 (1.30 - 3.67)	<0.001
Mean gradient (mmHg)	16 ± 5.2(7-28)	6 ± 2.28 (3-11)	<0.001
Systolic Pulmonary Artery Pressure (mmHg)	60.79 ± 9.7(40 – 85)	45.71 ± 11.2(30 – 70)	<0.001
Mitral regurgitation			
Non	31 (36.9%)	0	NS
Mild	51 (60.7%)	49 (58.3%)	
Moderate	2 (2.1%)	33 (39%)	
Severe	0	2 (2.1%)	
Wilkins score (mean ± SD) 8.4 ± 2.8			
Flexibility	3 ± 0.8		
Thickening	3 ± 0.6		
Sub-valvular	2 ± 0.5		
Calcification	1 ± 0.5		
NYHA Class			
I	0	56 (69.7%)	<0.001
II	0	26 (28.9%)	
III	59 (69.9%)	2 (2.6%)	
IV	25 (29.7%)	0	

Follow-up mitral valve area was 1.80 ± 0.49 (p-value <0.001), and mean gradient was 8 ± 4 mmHg (p-value 0.6). Post-procedural data, last follow-up, and clinical and echocardiographic data are shown in Table 2.

**Table 2:** Clinical and echocardiographic data of the patients after PTMC and during last follow-up. PTMC = Percutaneous transvenous mitral commissurotomy NYHA = New York Heart Association

	Post-PTMC	Follow up	P -value
Left atrial diameter (cm)	4.6 ± 0.7	4.7 ± 0.6	0.041
2D Mitral area (cm^2^)	1.83 ± 0.49 (1.10 - 3.50)	1.80 ± 0.49 (0.70 – 2.8)	<0.001
Doppler Mitral area (cm^2^)	1.94 ± 0.45 (1.30 - 3.67)	1.78 ± 0.53 (1.0 – 3.0)	0.475
Mean gradient (mmHg)	6 ± 2.28 (3-11)	8 ± 4 (4-15)	0.619
Systolic Pulmonary Artery Pressure (mmHg)	45.71 ± 11.2 (30 – 70)	40.03 ± 7.97 (30 – 55)	<0.001
Mitral regurgitation			
Non	0	0	0.086
Mild	49 (58.3%)	36 (42%)	
Moderate	33 (39%)	38 (45%)	
Severe	2 (2.1%)	10 (11.9%)	
NYHA Class			
I	56 (69.7%)	40 (51.2)	0.239
II	26 (28.9%)	20 (21.0)	
III	2 (2.6%)	14 (15.7)	
IV	0	10 (12.8)	
Mitral Restenosis		7 (8.3%)	

**Figure 1:**
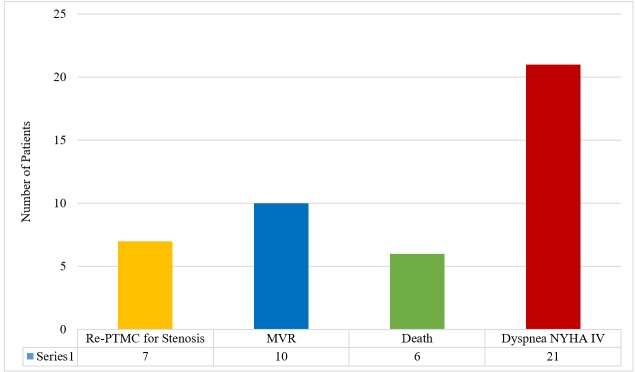
Events in patients in the follow-up period after PTMC PTMC = Percutaneous transvenous mitral commissurotomy MVR = Mitral valve replacement NYHA = New York Heart Association

Six deaths were recorded; four patients died from cardiac causes (two had severe persistent pulmonary hypertension, one had heart failure and one died of prosthetic value endocarditis), and two had non-cardiac cause of death. Mitral valve replacement was done in 10 patients (12.8%) due to severe mitral regurgitation, and re-PTMC was done for restenosis in seven patients (8.3%) in the follow-up period. Of the 21 patients who had deteriorated clinical conditions, 17 patients either underwent mitral valve replacement or re-PTMC, and four patients were thought to have deteriorated clinical condition due to persistent pulmonary hypertension (Figure 1).

MVA loss was considered for patients after taking the Doppler echocardiography MVA (at the six-months follow-up) as the baseline (since hemodynamics are stable at that time). The Doppler area calculated at 24 hours is not accurate due to acute changes in left atrial compliances. MVA loss was variable, with a mean area of 0.16 ± 0.11 cm^2^. When subgroup analysis was done according to Wilkin's score, it was similar in all subgroups (Table 3), with time (follow-up duration) as the only independent predictor of MVA loss.

**Table 3:** Mitral area in four subgroups of patients as defined by Wilkin's score before PTMC and during the follow-up PTMC = Percutaneous transvenous mitral commissurotomy MVA = Mitral valve area

Mitral Area (cm^2^)	4-6 cm^2^	7-8 cm^2^	8-10 cm^2^	≥11 cm^2^
No (%)	20(23)	42(50)	9(10)	3(4)
Baseline	1.2	1.0	0.9	0.85
Six months	2.1	1.78	1.62	1.39
Last follow up	1.89	1.52	1.42	1.26
MVA loss	0.21	0.26	0.20	0.13

Severe MR increased from 2.1% to 11.9%. When regurgitation was not severe, mild MR decreased by 16.3%, whereas moderate MR increased by 6% during the follow-up period. The major events recorded were restenosis and cardiac death. These events were plotted using Kaplan-Meier curve. Patients were divided in three groups according to the procedure performed: Group 1, restenosis; group 2, MVR; and group 3, death due to any cause.

**Figure 2:**
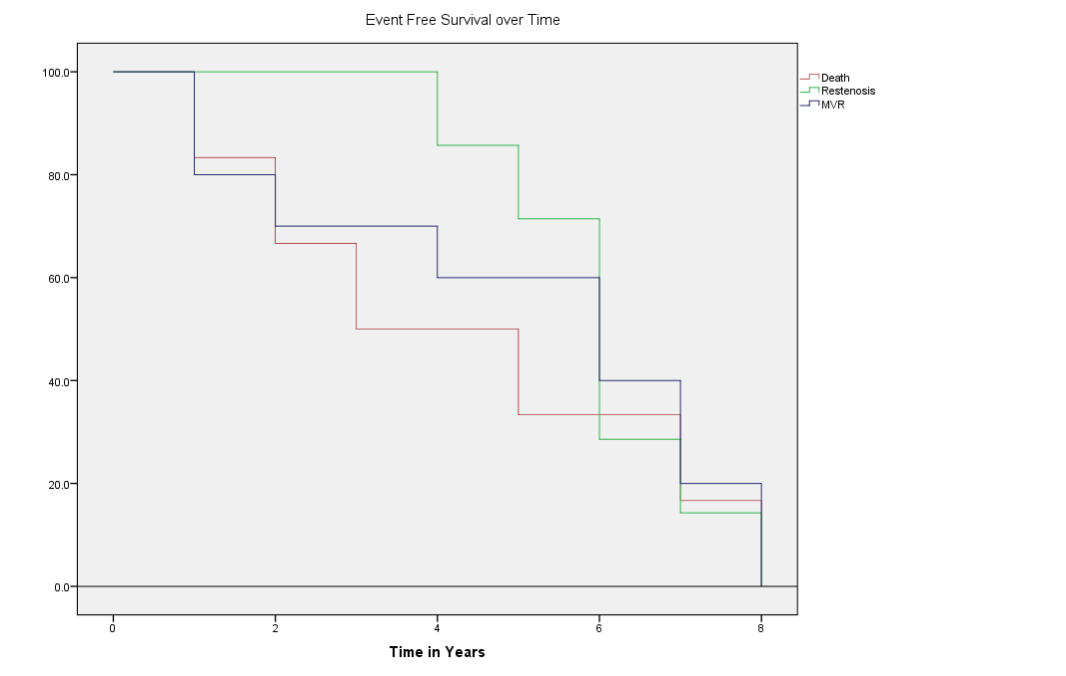
Event-free survival in patients over the follow-up period MVR = Mitral valve replacement

The curve shows that patients who presented with restenosis had better event-free survival than those who needed MVR, or than those who developed complications other than restenosis and died. It is evident from the curve that patients who require PTMC for mitral restenosis survive for a longer duration than patients requiring MVR, as well as those who had cardiac death due to severe pulmonary hypertension or heart failure. While longevity remains the same, the quality of life was much better in group 1. The curve shows that ultimately the survival is the same for all the three variables over eight years (Figure 2).

## Discussion

Percutaneous mitral valvuloplasty have developed significantly since its introduction in 1984. PTMC is indicated in symptomatic severe mitral stenosis because its short-term and long-term morbidity and mortality is good [6, 13-14]. The procedural and symptomatic outcome of PTMC is good and comparable with those of open surgical commissurotomy [15-16], but much better than the outcomes of close mitral valve commissurotomy procedures.

Our findings obtained in 100 patients successfully treated by PTMC showed an event-free survival of 70%. According to Bouleti, et al., the survival rate of successful PTMC of 20 years without intervention was 38% [9]. Post-PTMC, there was a significant clinical and hemodynamic improvement that was determined on long term follow-up.

The results obtained from our patients proved PTMC is a very valuable procedure in this era when rheumatic heart disease has declined in most of the developed countries. Our study involved PTMC in a population of mostly young women. The young age of the patients may be one reason for lower rate of complications and immediate good results.

There was an increase in the severity of mitral regurgitation in the follow-up period. Long-term prognostic factors after successful PTMC are reported in various studies [17]. Severe MR after PTMC is usually caused by a leaflet tear. It persisted during the follow-up, and 10 patients required mitral replacement in our study.

Mitral valve restenosis is found from 3%-50% within one to three years in patients who had successful PTMC [18-19]. Our data suggested that restenosis is rare within six years of the procedure in patients with a successful PTMC, but it increased over time, reaching 8.9% at eight years. In a post-PTMC series, Chen, et al. [14] reported an MVA decrease of 0.2 cm^2^ at five years, and Trevino, et al. [19] reported 0.25 cm^2^ at three years. In our series, MVA decreased to 0.16 ±0.11 cm^2^ at eight years, and was not affected by Wilkins score. Patients who had initial low area gain post-PTMC due to high scores had early MVA loss. Patients who were symptomatic in the long term were either due to restenosis, pulmonary hypertension, or severe MR. Cardiac death during follow-up was due to heart failure or severe pulmonary hypertension. Several studies are available regarding long-term follow up of PTMC, and have reported event-free survival ranging from 70-90% depending on the duration of the follow-up [20]. In our study, the event-free survival was of 70% at eight years. It is important to mention that in patients with favorable score and good MVA gain post-PTMC, the long-term survival is good. The patients who underwent PTMC and had restenosis in the long term even had a better survival for six years unless they had severe MR, severe pulmonary hypertension, or heart failure.

## Conclusions

Patients who undergo percutaneous transvenous mitral commissurotomy for symptomatic severe mitral stenosis had better, event-free survival. Those patients who had a favorable anatomy evaluated by Wilkin’s score had better short-term and long-term outcomes. The clinical and hemodynamic profiles were also improved in patients who underwent percutaneous transvenous mitral commissurotomy despite an unfavorable score.
